# Application of a specific clinical pathway can affect the choice of trial of labor in patients with a history of cesarean delivery

**DOI:** 10.1186/s12884-024-06429-8

**Published:** 2024-04-19

**Authors:** Petra Psenkova, Miroslav Tedla, Lenka Minarcinova, Jozef Zahumensky

**Affiliations:** 1 2nd Department of Gynecology and Obstetrics, University Hospital Bratislava and Comenius University, Ruzinovska 6, Bratislava, 82106 Slovakia; 2 Department of Otorhinolaryngology - Head and Neck Surgery, University Hospital Bratislava and Comenius University, Antolská 11, Bratislava, 851 07 Slovakia

**Keywords:** Frequency of cesarean delivery, History of cesarean delivery, TOLAC, Quality improvement, VBAC

## Abstract

**Background:**

Mode of delivery in women with previous history of cesarean delivery (CD) is highly modifiable by the practices of the delivery unit. Vaginal birth after a cesarean (VBAC) delivery is a safe and preferred alternative in most cases. The aim of this study was to assess the impact of adopting a complex set of measures aimed at the mode of delivery in this group.

**Methods:**

This was a retrospective observational study comparing two birth cohorts before and after the implementation of a series of quality improvement (QI) interventions. The study cohorts comprised women with a history of cesarean delivery who gave birth in the period before (January 2013 – December 2015) and after (January 2018 – December 2020) the adoption of the QI measures. The measures were focused on singleton term cephalic pregnancies with a low transverse incision in the uterus. Measures included approval of all planned CDs by a senior obstetrician, re-training staff on the use of the FIGO classification for intrapartum fetal cardiotocogram, establishing VBAC management guidelines, encouraging epidural analgesia during trial of labor after cesarean (TOLAC), establishing a labor ward team and introducing a monthly maternity audit.

**Results:**

Term singleton cephalic pregnancies with previous history of CD accounted for 12.55% of all births in the pre-intervention period and 12.01% in the post-intervention period. The frequency of cesarean deliveries decreased from 89.94% in the pre-intervention period to 64.47% in the post-intervention period (*p* < 0.0001). We observed a significant increase in TOLAC from 13.18 to 42.12% (*p*<0.0001) and also an increase in successful VBAC from 76.27 to 84.35% (*p* < 0.0001). All changes occurred without statistically significant change in overall perinatal mortality.

**Conclusions:**

This study demonstrates the feasibility to safely increase trial of labor and vaginal birth after cesarean delivery by implementing a series of quality improvement interventions and clinical pathway changes.

**Supplementary Information:**

The online version contains supplementary material available at 10.1186/s12884-024-06429-8.

## Background

The Slovak Republic is one of the countries in the European Union with the fastest increase in the frequency of cesarean deliveries in recent decades [1]. In 1998, 13.43% of women in the Slovak Republic gave birth by cesarean delivery (CD) and by 2019 this increased to 29.6% [2]. Currently, repeat CD accounts for approximately 25% of CDs overall [3], despite the established safety of vaginal birth after cesarean delivery (VBAC) in most cases. Thus, the mode of birth in women with previous history of CD is highly dependent on workplace practices [4]. There is consensus amongst several professional bodies (National Institute for Health and Care Excellence, Royal College of Obstetricians and Gynaecologists, American College of Obstetricians and Gynecologists [ACOG]) that planned VBAC is a clinically safe choice for the majority of women with a single previous lower segment caesarean delivery [5]. It is associated with fewer complications, lower risk of placenta previa and/or accreta in future pregnancies and pelvic adhesions compared to a planned cesarean delivery [5]. Hence, the American College of Obstetricians and Gynecologists (ACOG) recommends that a trial of labor after cesarean delivery (TOLAC) should be offered to all patients with a singleton pregnancy and a low transverse uterine incision [6].

The aim of this study was to assess the impact of adopting a complex set of measures aimed at the mode of delivery in this group. These measures involved the adoption of a new clinical pathway for the management of births in patients with a previous cesarean.

## Methods

We conducted a retrospective observational single-center study comparing two birth cohorts before and after the implementation of a series of quality improvement (QI) interventions. Therefore, our study reporting followed the Standards for Quality Improvement Reporting Excellence 2 (SQUIRE2) recommendations [7]. We included all women who gave birth in our hospital over two time periods - before (January 1, 2013, to December 31, 2015) and after (January 1, 2018 to December 21, 2020) the adoption of measures aimed at reducing cesarean deliveries. For the purpose of this study, we focused on term singleton cephalic pregnancies who previously had a CD.

The primary aim of the study was to assess if the adoption of a specific clinical pathway (QI intervention) can affect the mode of birth in patients with previous history of CD. We hypothesized that the adoption of these specific measures can lead to an increase in TOLAC and VBAC rates. As secondary outcomes were were interested to explore spontaneous labor and induction of labour rates. We also wanted to report on core neonatal outcomes, however, this information was only available for all births in our unit.

Irrespective of the QI interventions, the standard protocol for management of patients with a term singleton pregnancy and previous history of CD with a low transverse incision is to offer a TOLAC (unless vaginal delivery was contraindicated for other reasons). We allow the pregnancy to continue up to 10 days past the estimated date of delivery, as long as the general health of the mother and fetus allowed this. Patients are allowed to undergo induction of labor and are made aware that the preferred option is for labor to start spontaneously because of the associated higher success rate of vaginal birth and lower risk of uterine scar rupture. Patients with a fetal macrosomia are made aware of their reduced probability of achieving VBAC, nevertheless, not immediately denied a TOLAC. Patients with history of two previous CDs and a low transverse incision who request a TOLAC, are allowed to do as long as the labor commenced spontaneously by the estimated date of delivery.

The QI measures adopted to reduce repeat cesarean deliveries were part of a series of complex changes adopted in our clinic in 2016, which we previously reported [8]. The full list of measures is listed in supplemental table S1. Measures related to management of women with previous CD included organizational, staff training and unit policy measures as follows:

### • organizational

- Full time intrapartum care team on labor ward (the labor group) [9].
- Empowering midwives to manage labor, including the adoption of a one-to-one model of intrapartum care [10].

### • staff training

- Retraining maternity medical staff in intrapartum CTG interpretation and management based on FIGO classification [11].
- Evidence-based lectures for non-maternity medical staff about non-obstetric indications for CD [12].

### • unit policy

- Approval of elective indications for CD by the senior clinical management team, this measure also applied to private patients [13].
- Providing clinicians with clear recommendations and evidence-based information about TOLAC [14].
- Monthly audit and feedback of CD rates and indications [15].

To allow a “bedding in period” for the QI interventions to be fully incorporated in practice, we considered the time period between 2016 and 2017 as a transitional period and hence excluded from this analysis.

Data were collected from medical records. An anonymized database was then generated on Excel (Microsoft Excel version 2016) StatsDirect3 computer software was used for data analysis. Descriptive statistics were used, Student’s t-test (normally distributed data), Mann-Whitney U-test (non-normally distributed data) and Shapiro-Wilk test (determining normality) were used to evaluate the data. When comparing frequencies in the contingency table, the chi2-test was used. Data are presented as means and standard deviations (SD) or numbers and frequencies and unadjusted odds ratios (OR) as appropriate. The level of significance for all tests was set at 5%.

## Results

In the monitored periods, a total of 16,218 women gave birth in our unit, of whom 7,126 gave birth in the pre-intervention period (January 1, 2013, to December 31, 2015) and 9,092 in the post-intervention period (January 1, 2018, to December 31, 2020). Of these, 895 (12.56%) and 1092 (12.01%) were term singleton cephalic pregnancies with previous history of CD. Demographic details and birth outcomes of all births in our unit at both studied time periods are presented in Table 1.

**Table 1:** Demographic details and birth outcomes of total births in the studied periods

Parameter	Pre-intervention *N* = 7126	Post-intervention *N* = 9092	*p*
Mean Age years (SD)	31.37 (4.96)	31.68 (4.77)	< 0.0001
Mean birth weight in gm (SD)	3383 (452.29)	3448 (451.18)	< 0.0001
Spontaneous onset of Labor n (%)	4308 (60.45%)	6336 (69.69%)	< 0.0001
Induction of labor n (%)	1168 (16.39%)	1587 (17.45%)	0.07671
Epidural n (%)	2826 (39.66%)	4708 (51.78%)	< 0.0001
Spontaneous vaginal birth n (%)	4401 (61.78%)	6689 (73.57%)	< 0.0001
Operative vaginal birth n (%)	273 (3.83%)	581 (6.39%)	< 0.0001
Vacuum extraction n (%)	147 (2.06%)	509 (5.60%)	< 0.0001
Forceps delivery n (%)	126 (1.77%)	72 (0.79%)	< 0.0001
Total CD n (%)	2452 (34.41%)	1822 (20.04%)	< 0.0001
Planned CD n (%)	1650 (23.15%)	1169 (12.86%)	< 0.0001
History of CD n (%)	895 (12.56%)	1092 (12.01%)	0.29
Mild to moderate asphyxia^a^ n (%)	147 (2.04%)	102 (1.11%)	< 0.0001
Severe asphyxia^b^ n (%)	8 (0.11%)	19 (0.21%)	0.14
Perinatal mortality n (%)	18 (0.25%)	28 (0.31%)	0.5
Admissions to NICU n (%)	360 (5%)	417 (4.55%)	0.18

^a^ Mild to moderate asphyxia: Apgar score ≤ 3 at 1 min. and/or cord pH 7.0–7.1 + Apgar score ≥ 7 at 5 min.; ^b^ Severe asphyxia: Apgar ≤ 6 at 5 min. and/or cord Ph < 7.0. Explanations: NICU = neonatal intensive care unit. The Fisher test was used to calculate *p*-values

The percentages of term pregnancies with one fetus in the cephalic position and a history of CD were comparable in both of our studied cohorts (12.56% in the pre-intervention period and 12.01% in the post-intervention, *p* = 0.29).

There was a reduction in the frequency of CD performed in the post-intervention compared to the pre-intervention cohort (89.94% (805 of 895 births) to 64.47% (704 of 1092 births); (*p* < 0.0001). Furthermore, compared to the pre-intervention period, there was a reduction in planned CD (86.81% vs. 57.88%), increase in TOLAC (13.18% vs. 42.12%) and increase in VBAC (76.27% vs. 84.35%) rates in the post-intervention period. These differences were statistically significant (*p* < 0.0001) (Table 2). Furthermore, there was an increase in the proportions of women who had spontaneous onset (*n* = 376; 34.43% vs. *n* = 98; 10.95%; *p* < 0.0001) and induction ( *n* = 84; 7.69% vs. *n* = 20; 2.23%; *p* < 0.0001) of labor in the post-intervention group.

**Table 2:** Mode of birth outcomes in term singleton cephalic pregnancies with previous history of CD in the pre and post intervention periods

Birth outcome	Pre-intervention *N* = 895	Post-intervention *N* = 1092	OR (95%CI)	*P*
**All CD n (%)**	805 (89.94%)	704 (64.47%)	0.20 (0.16–0.26)	<0.0001
**Planned CD n (%)**	777 (86.81%)	632 (57.88%)	0.21 (0.17–0.26)	<0.0001
**TOLAC n (%)**	118 (13.18%)	460 (42.12%)	4.79 (3.82–6.03)	<0.0001
**VBAC n (%)**	90 (10.06%)	388 (35.53%)	4.93 (3.85–6.35)	<0.0001
**Spontaneous onset of Labor n (%)**	98 (10.95%)	376 (34.43)	-	<0.0001
**Induction of labor n (%)**	20 (2.23%)	84 (7.69%)	-	<0.0001

The Fisher test was used to calculate *p*-values

## Discussions

This retrospective cohort study has demonstrated a reduction in the number of CDs performed in term cephalic singleton pregnancies with previous history of a cesarean birth in a time period following the adoption and implementation of a set of interventions and policies. In the post-intervention period, there was an increase in the frequency of TOLAC and VBAC in this group. Our intervention consisted of several clinical and organizational changes. These changes included application of clinical pathways for the perinatal assessment and management of patients with a previous uterine scar. Furthermore, we empowered and supported staff by initiating a training program for CTG interpretation, creating a labor ward specialist team and extending the role of midwives within the unit. We believe that these changes helped and supported patients making informed choices about TOLAC and VBAC. When comparing the cohorts’ characteristics over the same time periods, the mean maternal age and mean birthweight were significantly increased in the post-intervention cohort. There was also an increase in overall operative vaginal delivery rates with a reduction in the number of forceps deliveries. Finally, there was a significant increase in epidural rates post-intervention (*p* < 0.0001).

The prominently different frequencies of CD in comparable units in the same country proves that mode of birth is influenced by clinicians and internal clinical pathways used in the management of pregnancy and delivery rather than patients’ demographics [4, 16, 17]. In our unit, the frequency of CD in the group of patients with a history of CD before the intervention was 89.94%. In this period, it was very unusual to give birth vaginally after cesarean. This could have been linked to traditional maternity care in our region. In 2016 we had a change in management within our unit. This facilitated the introduction of a series of interlinked changes aiming to increase the frequency of vaginal birth whilst ensuring maternal and fetal safety. These changes were facilitated, even enhanced, by the international drive and efforts of professional bodies involved in maternity care to achieve the same target, particularly in the context of TOLAC [5]. We also believe that there was strong public support to these changes within our region. It is also plausible that patients who were keen to have TOLAC selected our hospital over other neighboring units.

In our study, term singleton cephalic pregnancies with a previous CD accounted for 12.56% and 12.01% of all births in the pre-intervention and post-intervention periods respectively.This frequency tends to range between 5.2 and 30% [18, 19] in the published literature. According to Miller [20], the positive attitudes of patients and staff in the management of pregnancies following a CD increase the frequency of VBAC. Interestingly, maternal choice seems to vary throughout pregnancy with regards to mode of birth after CD. It was reported that at 24 weeks’ gestation, up to 85% of mothers considered TOLAC, nonetheless, only 40% actually remained committed to their decision by the time of delivery [21]. The rate of 40% of patients with previous CD opting for a TOLAC is comparable to the rate in our post-intervention cohort. Patients may lack the knowledge regarding the actual risks and benefits of TOLAC and planned CD. Moreover, if a patient feels that their provider has a preference, they are more likely to choose that mode of delivery [22]. In our study, providing consistent evidence-based information to patients about their individualized risks and supporting those who opted to give birth naturally after CD led to a significant increase in the number of mothers in the TOLAC group (from 13.18 to 42.12%, *p*<0.0001). This increase in TOLAC was also associated with and an increase in VBAC (from 76.27 to 84.35%, *p*<0.0001). Furthermore, counseling about mode of birth for patients with previous CD is undertaken by a senior clinician and followed by a written informed consent. We believe that providing patients and their birth partners with accurate information by a senior clinician and readiness to respond to any questions they had were the most crucial factors in increasing TOLAC in the group of women with a history of CD.

Rates of TOLAC vary between comparable units. In our region, there are 3 maternity units affiliated to the same university. A comparison of TOLAC rates between these units demonstrated that it ranged from 6.9% in one of these units to 30.3% in ours [4]. Similarly, success rate of VBAC varies between different reports. ACOG suggests that 60 to 80% of women who undergo TOLAC deliver vaginally [23]. However, in Singapore this rate was reported to be only 54.2% in a study conducted in 2021 [24], which only emphasizes the importance of adequate prenatal preparation of the mother and appropriate peripartum management. In 2017, the frequency of VBAC in our region was 44.8%, while it was 78.8% in our unit over the same time period [4], a rate similar to the 80% reported by a retrospective Australian study [25].

Factors that increase the probability of VBAC are a history of previous vaginal delivery and spontaneous onset of labor. Negative factors include a gestational age > 40 weeks, advanced maternal age, macrosomia, maternal obesity, incipient preeclampsia, and short inter-pregnancy interval [23, 26]. Therefore, adequate selection of patients suitable for TOLAC is crucial. Mothers should be made aware that the risk of rupture of the uterine scar increases with post-term pregnancy and labor induction. However, labor can still be induced if there was a clear maternal or fetal indication. The patient should be informed that there is a lower probability of VBAC with induction of labor than with spontaneous onset of contractions. According to a study conducted in France, in addition to BMI, the woman’s pre-existing diseases and suspected fetal macrosomia, the increasing age of the woman was a decisive factor in performing planned CD instead of attempting a vaginal delivery [27].

The group of women with a history of CD is characterized by its heterogeneity. It includes women who have a history of one or more cesarean deliveries. Additionally, these women may or may not have given birth vaginally in the past. Attempting a vaginal birth probably has a beneficial effect on the life-long health of the newborn [28]. Moreover, repeated CD entails several complications. The prevalence of intra-abdominal adhesions increases with each subsequent cesarean delivery. It has been reported that this risk is 32% after one, 42% after two and 59% after three or more CDs (*p* < 0.001) [29]. Furthermore, in a patient without a history of cesarean delivery, the risk of hysterectomy for abnormal placentation is 1:25.000 pregnancies, however, this risk increases to 1:500 and 1:20 with previous one and with 3 more previous CDs respectively [30]. In a study involving more than 771,000 mothers, Elvander and associates reported that patients who had a VBAC after one previous CD had a higher probability of giving birth to a third child compared to those who had two CDs [31]. Based on these results we can assume that by a VBAC, may increase the fertility rate of the given couple.

Due to the fact that in pregnancies with a previous CD, the mother’s request to end the delivery by CD is sufficient indication to perform it, proper and comprehensive information to the mother about the benefits and risks of both modes of delivery is extremely important [32]. In the pre-intervention period, 86.81% gave birth by planned cesarean delivery; in the post-intervention period, this decreased to 57.88% (*p* < 0.0001). According to Uddin, up to 85% of women after CD in the United States prefer a surgical abdominal delivery in their next pregnancy due to concerns about the risk of uterine rupture when attempting a vaginal delivery [33]. However, Bonzon et al. found that encouragement and recommendation of their doctor resulted in a fourfold increase in their likelihood of opting for a TOLAC [34].

Nevertheless, TOLAC is linked with a higher incidence of uterine rupture, neonatal asphyxia, and perinatal death compared to planned CD [35]. Hence, patients should be made fully aware of the risks, benefits, and alternatives connected to TOLAC and planned CD, which should be clearly documented and form part of the informed consent process. In order to ensure a good quality of provided healthcare, it is important to implement a standardized evidence-based protocol for the management of TOLAC. Providing the patient with a leaflet using clear, explicit, and unbiased language when presenting risks, benefits, alternatives, and related evidence associated with TOLAC would be also beneficial. The unit should also be equipped to deal with and resolve acute and potentially life-threatening complications related to TOLAC. Availability of multidisciplinary trained staff who can deal with emergencies if they happen and accessibility to an emergency operating room may not be readily available in small and community birth units. For these facilities, the distance to a referral unit with capabilities to deal with complications, if they arise, needs to be strictly evaluated. For units to ensure that patients get the best chance for a successful and safe TOLAC, it is imperative that the unit has facilities for continuous electronic fetal monitoring, staff that are up to date with their obstetric emergency drills and inter-professional skills training and have 24-hour access to an emergency operating room with experienced obstetric, anesthetic and neonatology teams [36, 37].

We recognize that the main limitation to our work lies in the retrospective nature of the study design. Indeed, this has limited our ability to explore more detailed information about demographic and clinical characteristics of the population, as well as, maternal morbidities, patient reported outcomes and core neonatal outcomes related to TOLAC. Nevertheless, the benefit of a retrospective, rather than a prospective, design in this context is that it mitigated the potential risk of bias secondary to a Hawthorne effect. Second, our data are generated from a single center and relate to a specific population who had a relatively high planned CD rate in the pre-intervention period and hence our findings might be perceived as not readily generalizable. Nonetheless, we believe that our study population is comparable to that of many maternity units in Europe with similar CD rates. However, the main strength to our work is that it demonstrates the potential for a series of feasible intervention to change practice and have a potential positive impact on mothers and their babies at the short and long-term.

## Conclusions

This study demonstrates the feasibility to safely increase trial of labor and vaginal birth after cesarean delivery rates by implementing a series of quality improvement interventions and clinical pathway changes. The increase in vaginal births did not seem to be associated with negative neonatal outcomes as our overall core neonatal outcomes rates were comparable before and after the adoption of these changes.

### Electronic supplementary material

Below is the link to the electronic supplementary material.

Supplementary Material 1

## Data Availability

The datasets used and/or analyzed during the current study are available from the corresponding author on reasonable request.

## Abbreviations

ACOG: American College of Obstetricians and Gynecologists
BMI: Body Mass Index
CD: cesarean delivery
SQUIRE 2: Standards for Quality Improvement Reporting Excellence 2
TOLAC: trial of labor after cesarean
VBAC: vaginal birth after cesarean
