# Supplementary material for: Application of a specific clinical pathway can affect the choice of trial of labor in patients with a history of cesarean delivery
Source: BMC Pregnancy Childbirth. 2024 Apr 19;24:292. doi: 10.1186/s12884-024-06429-8 (PMC11027349; doi:10.1186/s12884-024-06429-8)
Supplement: Supplementary file 1 — Supplementary Material 1 [file 12884_2024_6429_MOESM1_ESM.docx]

**Table S1: General list of quality improvement interventions and care pathway changes.**

| **Organizational** | Full time intrapartum care team on labor ward (the Labor ward specialist group). |
| --- | --- |
|  | Empowering midwives to manage labor, including the adoption of a one-to-one model of intrapartum care. |
| **Staff training** | Retraining maternity medical staff in intrapartum CTG interpretation and management. |
|  | Evidence-based lectures for non-maternity medical staff about non-obstetrical indications for CD. |
| **Unit Policy** | Omitting routine antepartal CTG monitoring from 36th week of gestation in low-risk pregnancies. |
|  | Approval of elective indications for CD by the senior clinical management team. |
|  | Providing clinicians with clear recommendations and evidence-based information about TOLAC. |
|  | Offering the external cephalic version to the pregnant patients with fetus in breech presentation. |
|  | Launching an induction of labor guideline including the implementation of Foley’s catheter into practice. |
|  | Offering the possibility of vaginal birth for women with a twin pregnancy and the first fetus in cephalic position. |
|  | Protocol for oxytocin use and dosage during labor. |
|  | Monthly audit and feedback of CD rates and indications. |

*Explanations*: *CTG=cardiotocogram, CD=cesarean delivery, TOLAC=trial of labor after cesarean*
